# Salinity Stress Mitigation in Durum Wheat via Seed Hormonal Priming

**DOI:** 10.3390/plants15071103

**Published:** 2026-04-03

**Authors:** Manel Hmissi, Khawla Nsiri, Rihab Zagoub, Vicente Gimeno-Nieves, Abdelmajid Krouma, Mohamed Chaieb, Francisco García-Sánchez

**Affiliations:** 1 Laboratory of Ecosystems and Biodiversity in Arid Land of Tunisia, Faculty of Sciences, University of Sfax, Sfax 3029, Tunisia; hmissimanel567@gmail.com (M.H.); khawlansiri206@gmail.com (K.N.); zagoubrihab123@gmail.com (R.Z.); abdelmajid.krouma@fstsbz.rnu.tn (A.K.); 2 Faculty of Sciences and Techniques of Sidi Bouzid, University of Kairouan, Kairouan 3100, Tunisia; mchaieb133@gmail.com; 3 Centro de Edafología y Biología Aplicada del Segura (CEBAS-CSIC), E-30100 Murcia, Spain; vgino@cebas.csic.es

**Keywords:** durum wheat, hormonal priming, chlorophyll fluorescence, ear filling, mean weight 100 grains, salinity

## Abstract

Seed priming is a simple, economical, and sustainable technique capable of enhancing crop resilience to abiotic stresses. A plastic greenhouse experiment was conducted on the durum wheat cultivar, Karim, sown in a 375 L volume container under semi-controlled conditions. Plots were arranged in a completely randomized design regarding treatments (control, salinity) and priming agents (indole-3-acetic acid, IAA; gibberellic acid, GA_3_; and salicylic acid, SA). Some physiological, biochemical, and morphometric traits were analyzed at vegetative and reproductive stages. The obtained results demonstrated that salinity stress reduced plant growth and the SPAD index, hampered photosynthetic efficiency through disrupted PSII integrity and energy management in the electron transfer chain, and significantly affected ear filling (EF) and grain caliber (marked by mean weight of 100 grains, MW100G). However, seed hormonal priming allowed the alleviation of salinity stress effects on durum wheat growth and yield. Although IAA and GA_3_ have shown significant potential in improving durum wheat tolerance to salinity, SA was found to be the most effective priming agent. It promotes the biosynthesis of chlorophyll pigments, restores the functional integrity of PSII, enhances photosynthetic efficiency, increases plant growth, and stimulates ear filling and wheat grain development. The principal component analysis demonstrated the interdependence of the vegetative and reproductive traits and presents SA as the most effective treatment that brings plants close to control conditions, despite the salinity.

## 1. Introduction

Plants interact with the environment, and all extreme conditions can cause stress and limit their growth and development. Certain species have the ability to develop different tolerance or adaptation mechanisms that enable them to defend themselves against stress factors. Global warming and climate change have intensified the impact of abiotic stress conditions on agricultural crops. Cereals are particularly sensitive to environmental stress factors. Consequently, there is an urgent need to adopt new farming practices in order to break the vicious circle between modern agricultural systems and climate change [1].

Despite the inherent ability of plants to adapt to environmental conditions, natural plant adaptation processes and increased genetic variability are unable to overcome the effects of rapid climate change and its collateral consequences. To address this problem, there has been an exponential increase in experimental studies in the laboratory, under semi-controlled conditions, and even in the field. These studies focus on abiotic stress factors, particularly drought, salinity, and temperature, with the aim of studying plant behavior at the morphological, physiological, biochemical, and molecular levels [2]. This is in order to identify tolerant species with high yields and high nutritional value. However, plant responses to abiotic stresses, in addition to their specificity, are also subject to varietal and/or genotypic changes and can cause reversible or irreversible changes in plant physiology and metabolism. These responses may also depend on the phenological stage, the intensity and duration of the stress, and the tissue or organ involved in the stress response mechanism.

Soil and water salinity is considered the main abiotic factor limiting crop productivity and agricultural yields worldwide. Some reports indicate that salinity reduces the area of irrigated land worldwide by 1 to 2% per year and continues to worsen in semi-arid and arid areas, where 25% of irrigated land is already salinized [3]. In Tunisia, salinity affects more than 1.5 million hectares, or about 10% of the total land area [4]. Climate change and the use of unconventional water for irrigation continue to exacerbate the problem. As a result, the gap between conventional water resources and irrigation demand has become significant. Soil and water salinity remains a major obstacle to plant growth in these regions. Salts accumulated in the soil can limit or completely halt plant growth due to an increase in osmotic pressure in the rhizosphere and/or the specific toxicity of certain elements [5]. In the Mediterranean region, salinity is a constraint in many areas of large-scale farming where water quality plays a major role and where the search for plants adapted to high salinity thresholds is becoming imperative for agricultural production. Varietal selection requires knowledge of the mechanisms responsible for plant tolerance to salinity.

To remedy the harmful effects of salinity and maintain a certain level of agricultural production in agrosystems subject to unavoidable salinity (saline soil and/or saline irrigation water), various approaches and techniques have been proposed. However, their effectiveness and sustainability remain the most critical points in the production chain. Salt stress tolerance is therefore a highly sought-after trait in agricultural species, in order to expand their cultivation in susceptible regions. The response of species to salt stress depends on genotypes, salt concentration, environmental conditions, and phenological stage, and is still far from being managed efficiently. Salinity generally has adverse effects at different stages of plant development cycle, including seed germination [6,7,8].

The adoption of effective and sustainable approaches to improve crop tolerance and mitigate the harmful effects of salinity remains the most promising approach. Seed pretreatment with organic, inorganic, or biological substances has been used for several decades to increase crop productivity under saline stress. In this context, various chemical and physical methods have been suggested, including hydro-priming, the use of nutrients, soaking in hormonal solutions, hydrochloric acid, sodium chloride, thioredoxin, sulfuric acid, seed scraping, etc. [9,10]. Indeed, various compounds have been tested to improve plant resilience to salinity stress, including salicylic acid, auxins, gibberellins, etc. Alternatively, seed pretreatment with such substances has gained ground as an alternative, low-cost approach to improving the germination potential of orthodox seeds and mitigating the effects of abiotic stress in various plant species [10,11]. Seed pretreatment has proven to be an effective means of improving, accelerating, and synchronizing seed germination, leading to better seedling establishment and crop growth, while breaking seed dormancy and preventing seed deterioration under unfavorable conditions to germination [7,8,11]. More importantly, recent scientific findings highlight that seed soaking induces tolerance to stress factors such as drought and salinity, thereby improving yield potential in various ecosystems [12,13,14]. The beneficial effects of seed priming have been reported for various crops, including squash [12,15], green bean [16], tomato [17], durum wheat [7,8], and *Sulla carnosa* [6].

Hormonal priming is increasingly recognized as an effective strategy to enhance seed germination and early seedling development under adverse environmental conditions [18]. This approach consists of pretreating seeds with specific phytohormones capable of regulating key physiological and biochemical pathways, thereby improving plant tolerance to stress. Several studies have highlighted the beneficial effects of hormonal priming with salicylic acid (SA) and gibberellic acid (GA) in promoting germination and early growth by modulating metabolic and defense-related processes [19,20,21].

GA is a major growth regulator involved in critical developmental events such as seed germination, stem elongation, leaf expansion, pollen development, and floral induction [22]. In cereal embryos, GA is synthesized and triggers the aleurone layer to produce hydrolytic enzymes, notably α-amylase, which mobilize endosperm reserves to supply nutrients required for embryo growth [23]. In addition, GA contributes significantly to plant adaptation to abiotic stresses, including salinity, by enhancing physiological performance under harsh conditions [22]. Otherwise, GA_3_ plays a crucial role in plant adaptive responses to abiotic stresses by promoting reserve mobilization and early developmental transitions, resulting in more vigorous germination and improved seedling establishment under adverse conditions. As a key regulator of hydrolytic enzyme activation, GA_3_ stimulates the breakdown of storage compounds such as starch, proteins, and lipids, thereby ensuring a rapid supply of metabolic energy and carbon skeletons for metabolic processes often hampered by salinity, drought, or heavy metals [24]. Studies have shown that priming with GA_3_ at concentrations of 50 to 100 mg L^−1^ increases germination by 20 to 35% compared to controls exposed to salt, while also improving seedling length by 25 to 40% [25,26]. Priming with GA_3_ (100 mg L^−1^) in wheat under 150 mM NaCl increased total chlorophyll content by 15–25% and improved the Fv/Fm ratio from 0.68 to 0.76 [26]. Otherwise, GA_3_ priming increased the number of grains per ear by 15–22%, the mean weight of 1000 grains by 10–18%, and the final yield by 12–25% under moderate salinity [25].

Indole-3-acetic acid (IAA), shown to be crucial for cell division and elongation, has also been effectively used in seed priming techniques to enhance the tolerance of crops to various stresses [18]. Pre-germination treatment has been suggested to promote salt tolerance and improve grain yield [25]. In this regard, priming *Triticum aestivum* grains with IAA mitigated the harmful effects of salinity by restoring germination, hormonal balance, and CO_2_ assimilation, leading to improved yields [25]. It has also been found that treating wheat seeds with IAA before sowing stimulated growth and photosynthetic activities under saline conditions [27]. This technique improved salt tolerance by modulating ion transport from roots to shoots [28]. In maize exposed to 120 mM NaCl, priming with IAA (10^−6^–10^−5^ M) resulted in a 28% increase in shoot biomass, a 32% improvement in root growth, and a 22% stimulation of α-amylase activity, promoting better mobilization of reserves [27]. IAA also contributes to maintaining chlorophyll fluorescence by activating antioxidant systems, with a reported 30% increase in superoxide dismutase activity and a 25% increase in catalase activity, thus limiting the accumulation of reactive oxygen species [27]. These improvements reflect better protection of thylakoid membranes and stabilization of photochemical processes. IAA, although less studied at the reproductive stage, showed an increase in ear dry weight of 12–17% under saline conditions, likely through improved assimilate transport and internal hormonal status [27].

Salicylic acid, another key phytohormone, plays a central role in plant defense and stress signaling [29] and is also involved in regulating germination under unfavorable environments [30]. SA enhances antioxidant activity and stabilizes cellular components, allowing seeds to maintain viability and germinate despite stress constraints. Salicylic acid (SA) is widely recognized as a central defense modulator that integrates environmental stress perception with transcriptional and metabolic reprogramming in plants. Although historically associated with pathogen-induced immunity and systemic acquired resistance (SAR), accumulating evidence demonstrates that SA also plays a critical role in plant responses to abiotic stresses such as drought, salinity, heat, and heavy metal toxicity. Under abiotic stress conditions, endogenous SA levels often increase, triggering NPR1-dependent and NPR1-independent signaling pathways that regulate stress-responsive gene expression, antioxidant defense systems, and osmoprotectant accumulation [31,32]. SA modulates reactive oxygen species (ROS) homeostasis by enhancing the activities of key antioxidant enzymes, including superoxide dismutase (SOD), catalase (CAT), and ascorbate peroxidase (APX), thereby limiting oxidative damage and stabilizing cellular membranes [33,34]. The application of 0.5–1 mM salicylic acid under water deficit (50% of field capacity) increased total dry matter by 18–30%, improved relative leaf water content by 12–20%, and reduced lipid peroxidation (MDA) by 25–40%, indicating improved membrane stability [35]. Salicylic acid (1 mM) restored the Fv/Fm ratio from 0.64 to 0.78 under water deficit and increased chlorophyll-a by 22% [33]. Salicylic acid, applied before sowing, improved grain filling by 18%, increased soluble sugar content by 20%, and reduced flower abortion by 15% under water stress [35]. Under salinity stress, exogenous application of SA improves vegetative growth by promoting ionic balance, reducing sodium accumulation, and maintaining adequate potassium levels, thereby limiting the toxic effects of salt on plant tissues [36]. Furthermore, salicylic acid (SA) protects chlorophyll pigments from salt stress-induced degradation. Recent work on early dwarf cashew has shown that SA increases chlorophyll content and enhances chlorophyll fluorescence, reflecting improved photosystem II efficiency and increased thylakoid membrane stability [37]. With regard to photosynthesis, SA stimulates stomatal conductance and CO_2_ fixation, thus contributing to the maintenance of high photosynthetic activity even in the presence of high NaCl concentrations. In maize, AS has been associated with the regulation of phytohormones and osmolytes, allowing the preservation of photosynthetic efficiency and the reduction in oxidative damage [38].

Given the proven effectiveness of hormones as priming agents, their specificity, and the increasing salinity of soil and irrigation water, even in irrigated areas [39], what physiological criteria should guide the choice of optimal priming protocols for durum wheat in Mediterranean saline agroecosystems? This study, therefore, aimed to evaluate the effect of hormonal seed priming on the physiological and morphometric characteristics of durum wheat subjected to salt stress—particularly on photosynthetic efficiency and yield components—and to answer several major questions: Which types of hormones best improve germination, growth, and yield of durum wheat under salinity stress? To what extent does hormonal priming contribute to preserving chlorophyll content and photosynthetic efficiency? And finally, what physiological mechanisms underlie salinity tolerance?

## 2. Results

It is important to note that at the end of the experiment, soil salinity measurements were made on all containers. On average, an increase in salinity was observed in soils irrigated with saline water (5 g L^−1^ NaCl), reaching 7.3 g L^−1^, while it remained around 2.1 g L^−1^ in the control soils.

### 2.1. Growth and Physiological Traits

**Figure 1:**
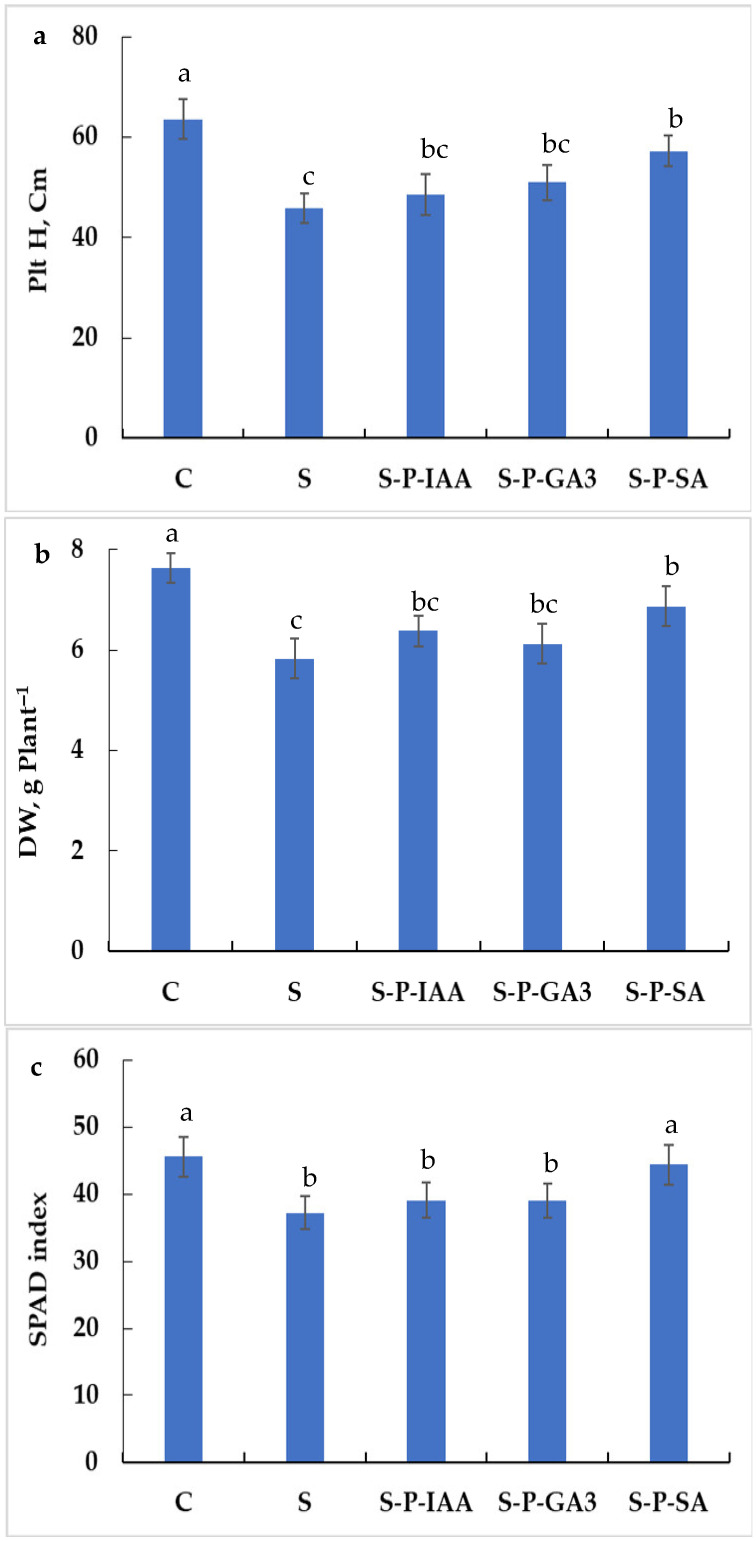
Plant height (**a**), dry biomass production (**b**), and SPAd index (**c**) in durum wheat plants subjected to salinity stress (5 g L^−1^ NaCl, S), control (0 NaCl, C), or subjected to salinity stress (5 g L^−1^ NaCl) but seeds were primed with indole 3-acetic acid (S-P-IAA), gibberellic acid (S-P-GA3), or salicylic acid (S-P-SA). Measurements are made 5 months after sowing. The significance of the differences is marked by the letters in the figures. Means with different letters are significantly different at α = 0.05 according to Fisher’s Least Significant Difference. The standard error of the mean of *n* = 30 plants (Three replicates of ten plants each) is represented by the bars on the columns.

When subjected to salinity stress, durum wheat plant height (Plt H) decreased by 28% (Figure 1a), whereas plant dry weight decreased by 24%, as compared to control plants (Figure 1b). Seed hormonal priming allows the improvement of growth parameters, depending on the used agent. Plant height decreased by 24%, 20%, and 10%, respectively, in IAA-, GA_3_-, and SA-primed plants, as compared to control (significant differences, *p* < 0.05; Figure 1a); whereas plant biomass decreased by 16%, 20%, and 10%, respectively, in IAA-, GA_3_-, and SA-primed plants, as compared to control (Figure 1b). When compared to the stressed condition, plant height increased by 6%, 11%, and 25%, respectively, in IAA-, GA_3_-, and SA-primed plants, whereas plant dry biomass increased by 9%, 5%, and 18%, respectively, in IAA-, GA_3_-, and SA-primed plants.

SPAD index is another parameter that explains the chlorophyll accumulation in plant leaves. Figure 1c shows that salinity stress significantly decreased SPAD index (−18%, as compared to control plants, Figure 1c), whereas seed priming with SA allows to alleviate this harmful effect on chlorophyll biosynthesis (SPAD index decreased by 3%, as compared to control plants; S-P-SA was not significantly different from C (*p* > 0.05), but significantly different from S, *p* < 0.05). The seed priming with IAA and GA3 does not show significant modification of SPAD index (S-P-IAA and S-P-GA3 were not significantly different from S, *p* > 0.05, but were significantly different from C, *p* < 0.05). When compared to stressed plants, the beneficial effect of SA seed priming is even more expressed (SPAD index increased by + 19% in S-P-SA plants, as compared to stressed non-primed plants, S).

**Figure 2:**
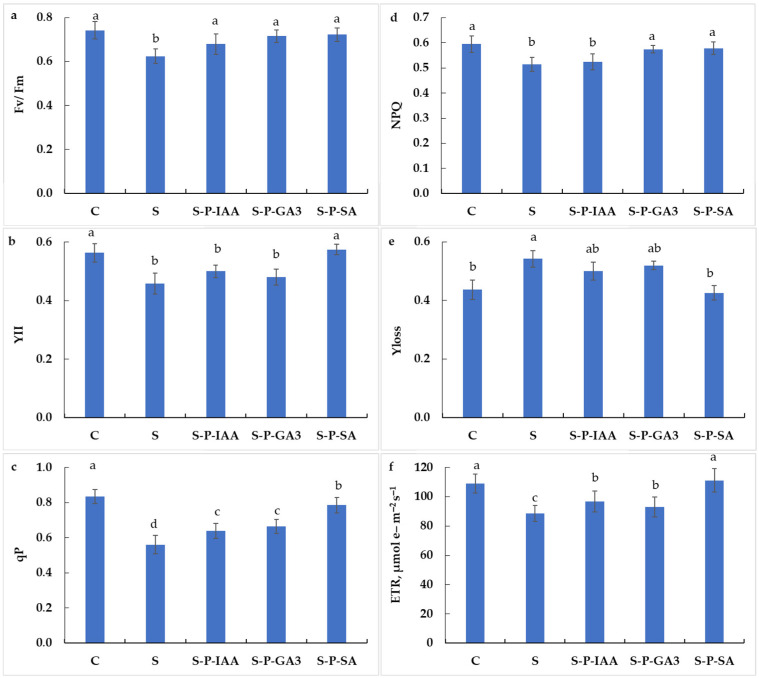
Maximum quantum yield (Fv/Fm, (**a**)), estimated photochemical efficiency index (YII, (**b**)), photochemical quenching (qP, (**c**)), non-photochemical quenching (NPQ, (**d**)), total energy loss (Yloss, (**e**)), and electron transport rate (ETR, (**f**)) in durum wheat plants subjected to salinity stress (5 g L^−1^ NaCl, S), control (0 NaCl, C), or subjected to salinity stress (5 g L^−1^ NaCl) but seeds were primed with indole 3-acetic acid (S-P-IAA), gibberellic acid (S-P-GA3), or salicylic acid (S-P-SA). Measurements are made 5 months after sowing). Measurements are made 5 months after sowing. The significance of the differences is marked by the letters in the figures. Means with different letters are significantly different at α = 0.05 according to Fisher’s Least Significant Difference. The standard error of the mean of *n* = 30 plants (Three replicates of ten plants each) is represented by the bars on the columns.

Figure 2a showed that salinity stress significantly hampered the maximum quantum yield of PSII (−16%, as compared to control plants), whereas seed hormonal priming clearly alleviated this negative effect of salinity. As compared to control plants, Fv/Fm decreased by 9% in S-P-IAA plants (allowing the recovery of 7% of Fv/Fm), 4% in S-P-GA3 plants (allowing the recovery of 12% of Fv/Fm), and 3% in S-P-SA plants (allowing the recovery of 13% of Fv/Fm, Figure 2a; differences not significant as compared to C, *p* > 0.05; and significant as compared to S, *p* < 0.05). The estimated photochemical efficiency index (YII, Figure 2b) does not escape this general behavior. Salinity stress significantly decreased YII (−19%, as compared to control plants, *p* < 0.05), whereas seed hormonal priming allowed the recovery of an important part of the lost YII under salinity stress (−11% in S-P-IAA plants, −15% in S-P-GA3 plants, and +2% in S-P-SA plants, instead of −19%, as compared to control plants). S-P-SA was not significantly different from C, *p* > 0.05.

The photochemical quenching exhibited a significant decrease under salinity stress, reaching −33%, as compared to non-stress conditions (*p* < 0.05, Figure 2c). However, seed priming with phytohormones allows to reduce this negative effect. Indeed, qP increased by 14%, 19%, and 40%, respectively, in S-P-IAA, S-P-GA3, and S-P-SA plants, as compared to S plants (Figure 2c). As compared to control plants, the qP decrease was reduced to −24%, −20%, and −6%, respectively, in S-P-IAA, S-P-GA3, and S-P-SA plants. For non-photochemical quenching, comparable behavior was also observed, with a less pronounced effect. As compared to control plants, NPQ decreased by 13%, 12%, 3%, and 3%, respectively, in S (significant difference from C, *p* < 0.05), S-P-IAA (significant difference from C, *p* < 0.05), S-P-GA_3_ (not significantly different from C, *p* > 0.05, but significantly different from S, *p* < 0.05), and S-P-SA plants (not significantly different from C, *p* > 0.05, but significantly different from S, *p* < 0.05) (Figure 2d). Under salinity stress, seed hormonal priming allowed them to recuperate 2 to 12% of NPQ, depending on the used hormone.

The total energy loss (Yloss, Figure 2e) and electron transport rate (ETR, Figure 2f) are other traits that explain the energy management in the electron transfer chain. Figure 2e shows that salinity stress significantly increased Yloss (+24%), whereas seed hormonal priming was able to cushion this effect, by reducing the energy loss (+14%, +19%, and −3%, respectively, in S-P-IAA, S-P-GA3, and S-P-SA plants (as compared to C plants, Figure 2e)). As compared to S plants, seed hormonal priming was able to decrease Yloss by 8%, 4%, and 21%, respectively, in S-P-IAA, S-P-GA_3_, and S-P-SA plants under salinity stress (differences are relatively significant for S-P-IAA and S-P-GA_3_ as compared to S, *p* < 0.05, and highly significant for S-P-SA as compared to S plants, *p* < 0.05; Figure 2e).

The ETR showed an opposite behavior to Yloss. Figure 2f showed that ETR decreased under salinity stress (−19%, −11%, and −15%, respectively, in S, S-P-IAA, S-P-GA3 plants, as compared to control plants), while remaining unchanged in S-P-SA plants. As compared to stressed plants, seed hormonal priming was able to increase ETR by 9%, 5%, and 25%, respectively, in S-P-IAA, S-P-GA3, and S-P-SA plants (Figure 2f).

### 2.2. Morphometric Traits

**Figure 3:**
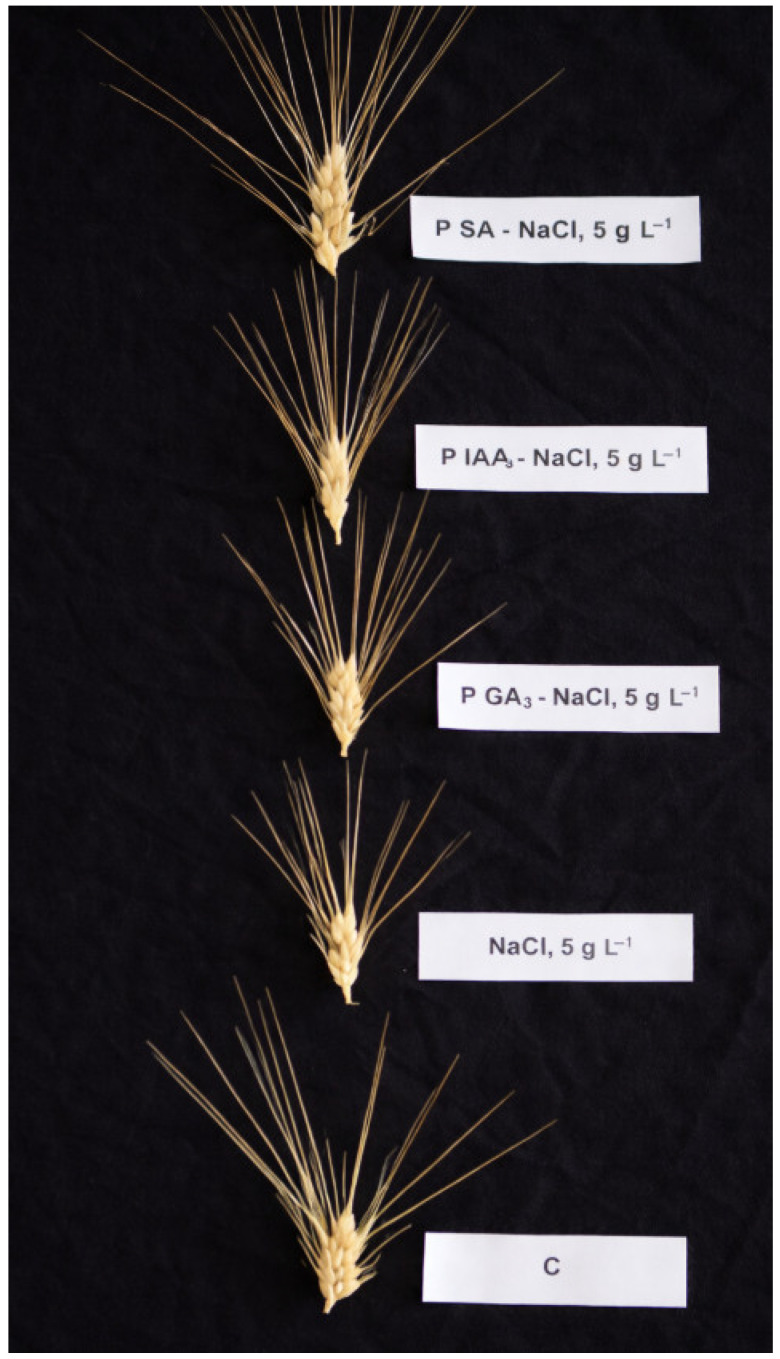
Ear morphology at maturation stage, in durum wheat plants subjected to salinity stress (5 g L^−1^ NaCl, S), control (0 NaCl, C), or subjected to salinity stress (5 g L^−1^ NaCl) but seeds were primed with indole 3-acetic acid (S-P-IAA), gibberellic acid (S-P-GA3), or salicylic acid (S-P-SA).

Plants were maintained until the completion of a full development cycle. Figure 3 illustrates the ear morphology and aspect before harvesting. Salinity stress resulted in reduced spike size. However, seed priming enhanced spike development under saline conditions, with salicylic acid (SA) showing the most significant effect among the evaluated hormonal treatments.

**Figure 4:**
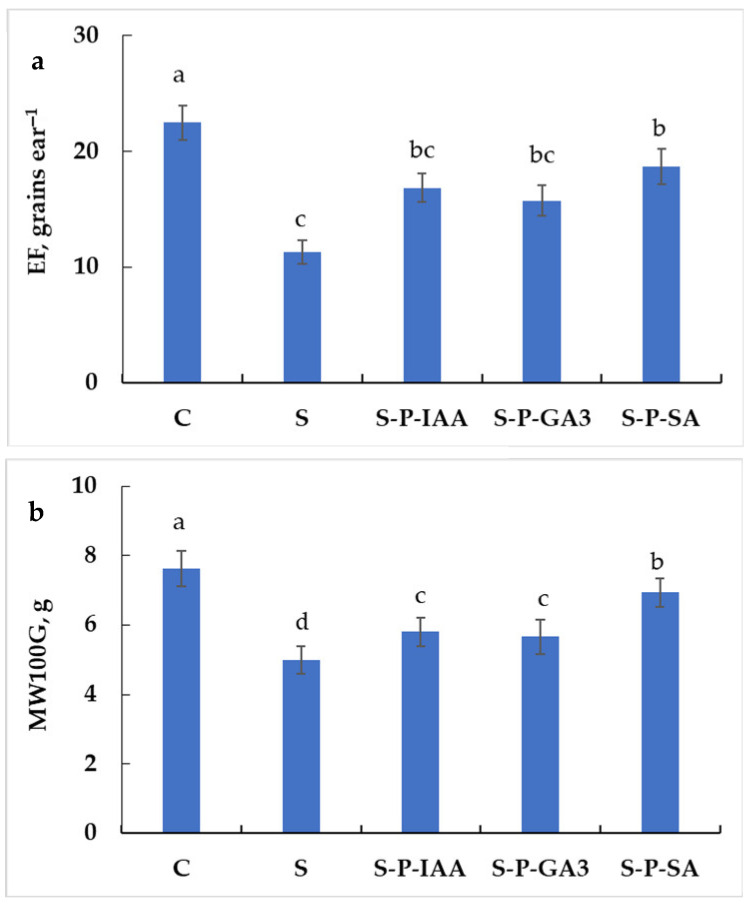
Ear filling (EF, (**a**)) and mean weight of 100 grains (**b**) in durum wheat plants subjected to salinity stress (5 g L^−1^ NaCl, S), control (0 NaCl, C), or subjected to salinity stress (5 g L^−1^ NaCl) but seeds were primed with indole 3-acetic acid (S-P-IAA), gibberellic acid (S-P-GA3), or salicylic acid (S-P-SA). Measurements were made at final harvest after ear ripening. The significance of the differences is marked by the letters in the figures. Means with different letters are significantly different at α = 0.05 according to Fisher’s Least Significant Difference. The standard error of the mean of 100 ears (five replicates of twenty ears) is represented by the bars on the columns.

To confirm the previous description of ears, we calculated the ear filling (EF, Figure 4a). This trait shows a significant decrease under salinity stress (−50%, as compared to control plants, shown in Figure 4a), whereas seed pretreatment with phytohormones was able to promote EF. The harmful effect of salinity declines to −25% in S-P-IAA plants, −30% in S-P-GA3 plants, and −17% in S-P-SA plants, as compared to C plants (Figure 4a). Indeed, the application of phytohormones as priming agents was able to increase ear filling by 49% when using IAA, 40% when using GA3, and 66% when using SA, under salinity stress.

The mean weight of 100 grains is another morphometric trait that reflects seed caliber. Figure 4b shows a significant decrease in this parameter under salinity stress, with a clear alleviation in seed primed plants. MW100G decreased by 34%, 24%, 26%, and 9%, respectively, in S, S-P-IAA, S-P-GA3, and S-P-SA plants, as compared to control plants (Figure 4b). When cultivated under salinity stress, seed hormonal priming was able to improve seed weight by 16% when using IAA, 13% when using GA3, and 39% when using SA, as compared to S plants.

In order to progress in the elucidation of the beneficial effect of seed hormonal priming and identify the better agent, we calculated the stress index based on the main measured vegetative and morphometric traits (plant height, SI-PltH; dry biomass, SI-DW; SPAD index, SI-Spad; Fv/Fm, SI-Fv/Fm; ear filling, SE-EF; and mean weight of 100 grains, SI-MW100G). Table 1 shows that hormonal priming decreased SI-PltH by 16% when using IAA, 29% when using GA3, and 64% when using SA; SI-DW by 31% when using IAA, 16% when using GA_3_, and 58% when using SA; SI-SPAD by 22% when using IAA, 21% when using GA_3_, and 86% when using SA; SI-Fv/Fm by 46% when using IAA, 77% when using GA_3_, and 83% when using SA; SI-EF by 50% when using IAA, 40% when using GA_3_, and 66% when using SA; and SI-MW100G by 31% when using IAA, 25% when using GA_3_, and 74% when using SA. All treatments were significantly different from C, with the most important significance being in S-P-SA, *p* < 0.05.

Regarding all the used hormonal agents and all calculated traits, SA showed the highest effectiveness, with the lowest SI values.

Finally, to further explore the relationship between the different functional traits analyzed at the vegetative and reproductive levels, and to identify the common thread between these parameters, we conducted a principal component analysis (PCA). Table 2, which illustrates the Pearson correlation matrix, shows a positive and highly strict correlation between growth parameters (Plt-H and DW) and chlorophyll fluorescence parameters that illustrate the photosynthetic performance (Fv/Fm, YII, ETR), between growth (Plt H and DW) and morphometric parameters (EF, MW100G), and between fluorescence and morphometric parameters.

**Figure 5:**
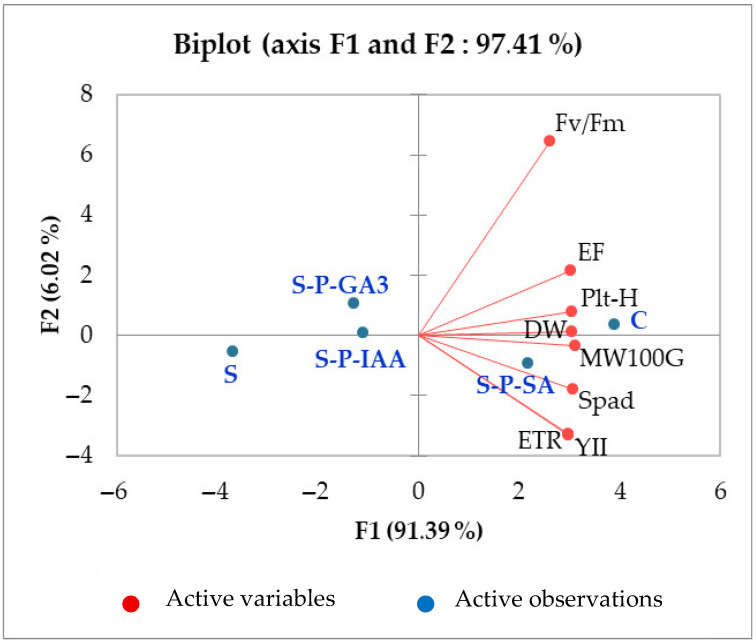
Biplot presentation of active variables (DW, EF, ETR, Fv/Fm, MW100G, Plt-H, Spad, YII) and active observations (C, S, S-P-GA_3_, S-P-IAA, S-P, SA) in durum wheat plants subjected to salinity stress (5 g L^−1^ NaCl, S), not subjected to salinity stress (C), or subjected to salinity stress (5 g L^−1^ NaCl) but seeds were primed with indole 3-acetic acid (S-P-IAA), gibberellic acid (S-P-GA_3_), or salicylic acid (S-P-SA).

The biplot generated from the PCA (Figure 5) presented two types of information. It explains 97.41% of the active variables (in red), with 91.39% for axis F1. This means that most of the information is contained in axis F1. Otherwise, all presented parameters are located on the positive side of the F1 axis, which confirms their strong correlations and interdependence. The second type of information is the active observations related to treatments (in blue). Their position explains their relationships with the active variables. For example, C (control) is close to the variables on the right, meaning that this observation has high values for Fv/Fm, EF, DW, Spad, etc., whereas S (stress) is on the left (negative on F1), meaning that this observation has low values for these variables. S-P-SA is on the right but at the bottom; it is highly correlated with ETR and YII. Indeed, F1 seems to represent a gradient of physiological performance or growth (the variables chlorophyll fluorescence and plant growth are correlated). F2 slightly distinguishes the treatments according to variables such as Fv/Fm (high) and ETR/YII (low). C is the most favorable treatment (strong correlation with all variables). S is the least favorable (low values for all variables). S-P-SA remains the closest to C on F1, which explains 91.39 of the obtained results (active variables), which could suggest a better performance compared to the other treatments. For inter-trait correlations, a strong positive correlation is observed between ETR, Y(II), SPAD, DW, and MW100G. These vectors are closely aligned and oriented in the same direction along the positive side of F1. This indicates that higher chlorophyll content (SPAD), improved photochemical efficiency under light (YII), and higher electron transport rate (ETR) are associated with greater dry weight accumulation and increased MW100G. Physiologically, this suggests that improved photosynthetic performance translates directly into enhanced biomass production and grain filling. EF and Plt-H (plant height) also cluster in the same quadrant, showing positive correlations with biomass and yield-related traits, although their vectors are slightly separated, suggesting moderate rather than very strong correlations.

## 3. Discussion

Throughout their life cycle, cereal crops are constantly exposed to abiotic stresses, among them salinity, that significantly hamper their growth and productivity, posing a serious threat to global food security [40]. In the present study, salinity stress significantly decreased durum wheat plant growth and SPAD index, and disrupted PSII quantum yield and energy management, whereas seed hormonal priming allowed the alleviation of stress effects, depending on the used priming agent. Salicylic acid has proven to be the most effective priming candidate, followed by GA_3_ and IAA, respectively. Despite their diversity and adaptability to bioclimatic conditions, cereals, and especially durum wheat, remain highly sensitive to abiotic stress, while the situation has worsened with climate change. Hossain et al. [40] reported that salinity stress significantly limits crop growth, yield, and quality, posing a serious threat to global food production. In cereal crops, salinity disrupts the key physiological processes such as photosynthesis, nutrient uptake, respiration, and reproduction, ultimately reducing productivity [41]. In fact, growth reduction is one of the first responses to salinity [42] known to occur in all plants and varies widely among species and varieties within a species, depending on the developmental stage at which salinity is initiated, the duration of exposure to salt stress, the salt concentration, the salt composition, and interactions with the environment. It results in a considerable reduction in the number of leaves, fresh weight, and the dry weight of the leaves, stems, and roots [43]. In *Melilotus segetalis*, 170 mM NaCl inhibits vegetative growth and reduces leaf biomass and the efficiency of these organs in using nutrients (K, Ca) for biomass production [44]. It also modifies the distribution of dry matter and nutrients among the organs. These effects depend on the severity of the stress, duration of treatments, and the age of the plant. As observed in the present study, chlorophyll fluorescence and photosynthetic efficiency are not exempt from these harmful effects of salinity. In soybean, Feng et al. [45] demonstrated that salinity has a very significant negative effect on the absorption of light energy during photosynthesis, accelerating the senescence process and decreasing biomass production, and they attributed the inhibition of photosynthesis under salt stress to reduced stomatal conductance. Nevertheless, plant growth regulators are known small endogenous signaling molecules, including auxin, gibberellins, cytokinin, abscisic acid (ABA), ethylene, brassinosteroids, salicylic acid (SA), and jasmonic acid (JA). Among these hormones, auxin and gibberellin are known to play a major role in plant developmental responses, while SA is involved in modulating plant defense responses against biotic and abiotic stresses [46]. Seed priming with hormonal solutions plays an important role in seed metabolism with positive repercussions at the different phenological stages [47]. It is a commonly used technique to improve seed germination, seedling growth, and crop yield under adverse conditions [48,49]. Several studies have shown that pretreating seeds with an optimal concentration of phytohormone improves germination, seedling growth, and yield by enhancing nutrient uptake through improved physiological activities and root production [50,51]. These beneficial effects of phytohormones have been demonstrated in several cultivated species where they regulate numerous physiological processes such as growth and development, respiration, and transpiration [52,53,54].

It has been widely documented that analyzing changes in chlorophyll fluorescence can be used to study the effects of different stresses on photosynthesis as well as the absorption and use of light energy by PSII. In this context and with a total agreement with the present results, Feng et al. [45] demonstrated that Fv/Fm represents the photoinhibition index and reflects the efficiency of light energy conversion by PSII, significantly decreased in the salt-sensitive soybean genotype, without being altered in the tolerant genotype, subjected to salt stress. The same authors [45] state that the PSII of sensitive genotypes is more sensitive to salt stress than that of tolerant genotypes, and they confirm that salinity causes damage to the photosynthetic apparatus and inhibits the electron transport chain, which explains the decrease in net photosynthetic assimilation. Indeed, the preservation of PSII remains dependent on the proper management of energy from incident light. This management is ensured by quenching, which encompasses a set of mechanisms by which the light energy absorbed by pigments (notably chlorophyll) is dissipated or regulated to prevent damage to photosynthetic systems. Photochemical quenching (qP) represents the excited energy used for photochemical reactions (electron production in PSII). In this study, it decreased under salt stress and increased significantly following the application of hormones, especially SA. Non-photochemical quenching (NPQ) represents the excess energy dissipated as heat or through other pathways not directly related to photochemical conversion. Instead of increasing, it decreased under stress, but increased in seed-primed plants, especially S-P-SA plants. In fact, NPQ is a protective mechanism that prevents damage caused by excessive light [55]. It is considered a heat dissipation mechanism and depends on several components: energy-dependent (qE), zeaxanthin-dependent (qZ), and photoinhibitory quenching (qI) [56]. The first two components are essential for photoprotection, while qI could represent the photoinhibitory damage to PSII reaction centers [57]. Thus, the decrease in NPQ observed in this study under salt stress could be explained, as demonstrated by Baker [58], by inactivation and/or degradation of D1 proteins leading to PSII failure, or to disruption of the xanthophyll cycle. Accordingly, Lauterberg et al. [59] reported in chickpea (*Cicer arietinum*) that low tissue hydration under water deficit reduces membrane flexibility and disrupts the xanthophyll cycle, leading to a decrease in NPQ, correlated with a decrease in yield. Under salt stress, Zuo et al. [60] showed that exposing rice (*Oryza sativa*) plants to high salinity leads to a reduction in NPQ, reflecting a decrease in photoprotective capacity. Salt-sensitive varieties exhibit a more pronounced drop in NPQ, accompanied by an increase in photoinhibition and the production of reactive oxygen species (ROS). However, the present results demonstrated that hormonal priming of wheat seeds, primarily GA_3_ and SA, leads to an increase in NPQ close to the control values. This reflects their beneficial effect on preserving the structure and functionality of PSII and its various components, perhaps through protection against ROS or toxic Na^+^ ions, or through improved dissipation of excess light energy as heat. Accordingly, Khalid et al. [61] demonstrated that applying seaweed extracts to tomato and maize under salt or water stress leads to an increase in NPQ, correlated with improved tolerance and reduced photoinhibition.

Nevertheless, the present study investigated the effect of salinity on the morphometric parameters of durum wheat at the reproductive stage and highlighted the beneficial effect of seed hormonal priming on the produced ears and grains. The harmful effect of salinity on ear length and morphology, ear filling, and grain caliber (MW100G) was confirmed, alongside the beneficial effect of seed hormonal priming, depending on the used agent. At this level, SA gained its superiority over the other phytohormones. The reduction in ear size and the number of grains per ear (EF) observed in this study under salinity stress can be explained by a high abortion rate at flowering stage, while the decrease in MW100G could be explained by the restriction of grain supply by organic matter from photosynthesis. This is entirely in line with the reduction in photosynthetic efficiency observed in this study. The improvement in these morphometric traits in hormonal-primed seeds plants could be explained by the promoted photosynthetic efficiency attributed to the restoration of PSII structure and performance, and energy management. In addition, the effectiveness of certain of these hormonal agents could be attributed to the 49%, 40%, and 66% increase in EF; 16%, 13%; and 39% increase in MW100G; and 9%, 5%, and 25% increase in the estimated photochemical efficiency index (YII), respectively, in IAA, GA_3_, and SA primed plants. Accordingly, Suo et al. [62] demonstrated that salt stress also has harmful effects on plant reproductive organs, leading to altered microsporogenesis, inhibition of stamen filament elongation, ovule abortion, and premature embryo aging. However, salicylic acid has been shown to act as a stimulator of flowering in plants subjected to salt stress [63]. Naeem et al. [64], Torres Mendonça et al. [65], and Lacerda et al. [66] demonstrated that salt stress leads to a reduction in fruit diameter in tomato, okra, and guava. This phenomenon can be attributed to the harmful effects of salt stress on gas exchange and the limited translocation of photoassimilates, mainly due to difficulties in nutrient and water absorption.

The functional dissection of the modes of action of GA_3_, IAA, and SA, used as priming agents for durum wheat seeds have revealed differences in action. While our physiological data suggest persistent benefits of SA priming, future studies incorporating molecular markers could test whether epigenetic modifications contribute to this stress memory. The beneficial effect of hormonal seed priming constitutes a strong asset for suggesting an increase in durum wheat tolerance to salinity. Gibberellins are growth hormones with confirmed positive effects on seed germination, shoot elongation, flowering initiation, and flower and fruit development, thereby regulating plant growth and development throughout their life cycle [18]. For the beneficial effect of GA_3_ observed in this study, numerous studies have demonstrated its involvement in the plant’s response to salt stress and in countering the harmful effects of stress. Moreover, the application of GA_3_ under saline conditions mitigated the harmful effect of Na^+^ on growth. Several authors noted that GA_3_ in saline environments improves growth and provides resistance to salinity [52,67,68]. Janah et al. [69] reported that GA appears to boost salt tolerance by regulating Na^+^/H^+^ antiporter genes and activating salt-responsive proteins that help maintain osmotic balance. The same authors [69] demonstrated that priming with SA and GA significantly enhances growth parameters, including plumule length, radicle length, and seedling dry weight. Sghayer et al. [70] reported that Na^+^ accumulation in seedling tissues significantly impaired carbohydrate and protein mobilization by inhibiting amylase and protease activities, but had lesser effects on primed seeds. This underscores the potential of SA and GA as effective priming agents for promoting seedling growth in challenging environments. Indeed, our results suggest that GA_3_, which ranks second after SA in terms of effectiveness, stimulates certain metabolic functions in the seed, such as respiration and biosynthesis of growth hormones, and reduces abscisic acid levels, having positive effects at later stages of the development cycle, including the yield and quality of the produced ears and grains. In line with our results, Iqbal and Ashraf [28] emphasized that seed priming with IAA enhances plant resistance to salt stress through the modulation of ion homeostasis in wheat and induces the biosynthesis of salicylic acid in the leaves. Other studies showed that IAA is the first-identified and best-known phytohormone, which plays a crucial role in modulating plant growth and developmental processes, such as root growth, cell elongation, vascular differentiation, and apical dominance [71]. Nonetheless, the available literature indicates that SA seed priming effectively enhances plant resilience to various abiotic stresses [72,73,74,75,76,77,78,79]. The exceptional prominence of SA in this study was recently described by Barbosa et al. [80], who demonstrated its beneficial effect on the photosynthetic apparatus and gas exchange in *Tropaeolum majus*. Khan et al. [81] reported that the application of low concentrations of SA could provide salinity stress tolerance by improving physiological processes and enhancing salt tolerance. In *Arabidopsis* and tomato, SA positively regulates salt tolerance [82], whereas in rice and *Cucurbita pepo*, it plays a negative role in the response to salt stress [83,84]. Other authors reported that seed priming with low concentrations of SA not only promoted seed tolerance to salinity but also enabled faster recovery of growth after emergence [70]. Under salt stress conditions, SA helps to mitigate the adverse effects of stress by stabilizing cell membranes, enhancing antioxidant enzyme activity, and reducing oxidative stress, thereby supporting better early seedling development and establishment. Indeed, it is suggested that these differences in response to biostimulants, sometimes contradictory, are linked to the specificity of the response to salt stress, on the one hand, and to the variability of the reaction to biostimulants, on the other hand, without neglecting the method of application of exogenous agents (priming, spraying, or direct application in the rhizosphere). Our findings are consistent with the conclusions reported by Karlidag et al. [85], Yildirim et al. [86], and El-Hady et al. [87]. They demonstrated that the application of salicylic acid led to an increase in dry-matter content of *Fragaria ananassa*, *Cucumis sativus*, and *Solanum lycopersicum*, and attributed this response to the induction of antioxidant responses and the protective role of membranes, thereby enhancing the plant’s ability to tolerate stress. Other authors [88] demonstrated that SA stimulated carbon dioxide (CO_2_) assimilation and photosynthesis rates, resulting in increased dry matter production, and attributed this response to better mineral uptake by plants subjected to stress. The improved fluorescence parameters observed in this study are also in accordance with those of Moustakas et al. [89]. As demonstrated in other species [90], the beneficial effect of SA may reflect its strong ability to regulate various defense mechanisms, particularly maintaining ionic balance, sustaining photosynthetic activity, and detoxifying reactive oxygen species. The upcoming studies planned within this research framework, which will focus on ion compartmentalization (K/Na exchange), membrane stability, the production of reactive oxygen species, and antioxidant capacity (APX, CAT, POD, SOD), will provide answers at this level regarding the durum wheat–salinity–SA interaction. Presumably, SA plays a crucial role in modulating the antioxidant machinery under salinity stress, when applied at optimal concentrations, by enhancing the activities of antioxidant enzymes, thereby improving ROS scavenging capacity and maintaining redox balance [33,91]. This effect is partly mediated by SA-induced accumulation of H_2_O_2_, which functions as a signaling molecule to activate defense-related genes and antioxidant responses [92,93]. Moreover, as a phytohormone, SA plays a crucial role in hormonal crosstalk under saline stress conditions. In this regard, Torun et al. [94] demonstrated that SA can efficiently regulate hormone production in plants under saline stress by promoting auxin production, while reducing the production of other hormones such as ABA and ethylene. Auxin (AIA) is recognized for its essential role in cell division and elongation, while ABA and ethylene generally inhibit plant growth under saline conditions [95]. By regulating hormone levels, SA enhances the plant’s ability to develop.

**Figure 6:**
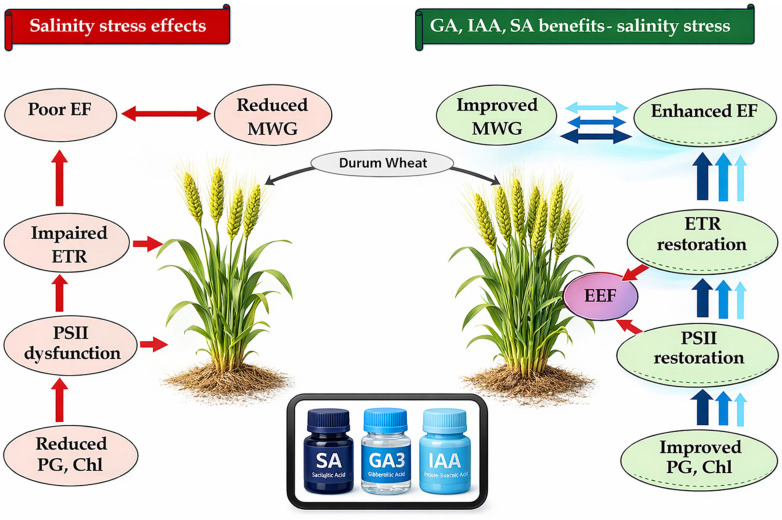
Summary diagram of the mechanism of hormones’ action as priming agents for durum wheat response to salinity stress. Chl: Chlorophyll; EEF: efficient energy flow; EF: ear filling; ETR: electron transfer rate; MWG: mean weight of grains; PG: plant growth. Dark blue arrow: High efficiency; sky blue arrow: moderate efficiency; light blue arrow: lowest efficiency. “Proposed mechanisms are based on observed physiological correlations; direct molecular validation is planned for future work.”

Finally, the designed PCA and calculated stress index (SI), regarding the applied treatments and evaluated traits, clearly discriminated between the applied treatments and the used hormonal solutions. Although IAA and GA_3_ have shown significant potential in improving durum wheat resilience to salinity, SA was found to be the most effective priming agent, followed by GA_3_ and IAA. SA promotes the biosynthesis of chlorophyll pigments, restores the functional integrity of PSII, enhances photosynthetic efficiency, increases plant growth, and stimulates ear filling and wheat grain development. The PCA demonstrated the interdependence of vegetative and reproductive traits and presents SA as the most effective treatment that brings plants close to control conditions, despite the salinity. Figure 6 summarizes the mechanism of action of hormones as priming agents for durum wheat seeds on key metabolic functions during the vegetative and reproductive stages.

## 4. Materials and Methods

### 4.1. Plant Material and Experimental Design

The durum wheat cultivar Karim, provided to us by the National Institute of Field Crops in Bousalem (INGC; Tunisia), is used in this study.

To assess the importance of seed priming on the overall physiological, biochemical, and morphometric traits at vegetative and reproductive stages of the plant cycle, uniform and healthy seeds were surface-cleaned and subjected to hormonal priming treatments. Seeds were soaked in freshly prepared aqueous solutions of gibberellic acid (GA_3_), indole-3-acetic acid (IAA), or salicylic acid (SA) at a concentration of 1 mM. The soaking was performed for 6 h at room temperature (approximately 22–25 °C), under gentle agitation to ensure homogeneous imbibition and uniform exposure to the priming solutions. The choice of 6 h is based on laboratory optimization trials (not yet published) and the literature [96,97]. The volume of each solution is adjusted so that the seeds are completely submerged.

After priming, seeds were removed from the solutions and gently blotted with absorbent paper to remove excess surface moisture. They were then air-dried (dehydrated) at room temperature for 48 h until reaching approximately their original moisture content, ensuring that metabolic activation induced by priming was maintained without initiating radicle protrusion. Dried seeds were subsequently stored under dry conditions at room temperature until sowing.

To begin, a greenhouse experiment was conducted in the experimental plot of the Faculty of Sciences and Techniques of Sidi Bouzid (Long. 9.4839392; Lat. 35.0354386) during the 2024 season. The experimental structure consisted of a greenhouse covered with a transparent plastic film only on the upper part to prevent direct rainfall, while the sides remained completely open to maintain natural ambient temperature and humidity conditions. This setup allowed for a controlled water supply while maintaining a microclimate close to open-field conditions. The average temperatures recorded during the trial were 22–28 °C (day) and 11–16 °C (night) in autumn, 14–17 °C (day) and 4–7 °C (night) in winter, and 20–26 °C (day) and 9–14 °C (night) in spring. The average relative humidity ranged from 50–60% in autumn to 65–75% in winter and 55–65% in spring. Plants were grown under natural light conditions, with an average monthly sunshine duration of approximately 8.68 h in October (sowing period), 7.77 h in December (the lowest recorded value), and 13.36 h in May (harvest period). All climatic data were provided by the Sidi Bouzid meteorological station, located approximately 100 m from the experimental plot, ensuring accurate representation of local environmental conditions.

Seeds were sown directly into soil-filled containers (2 m^2^ surface area) at a density of 400 seeds m^−2^ (800 seeds per container). The main soil characteristics are as follows: pH: 7.03; organic matter: 1.33%; active lime: 5.0%; total carbonates: 6.48%; Fe: 0.51%; K: 1.125%; Mg: 0.60%; N: 0.71%; C: 0.59%; and P: 0.15%.

The container constituted the experimental unit, as treatments were applied uniformly at the container level. Individual plants within each container were considered subsamples. Five treatments were established: (i) control (C; irrigated with tap water), (ii) salinity stress (S; irrigated with 5 g L^−1^ NaCl), (iii) salinity + IAA-primed seeds (S-P-IAA), (iv) salinity + GA_3_-primed seeds (S-P-GA_3_), and (v) salinity + SA-primed seeds (S-P-SA). Each treatment was replicated three times, resulting in a total of 15 containers. The containers were thoroughly irrigated to facilitate soil leaching and percolation of excess water, thereby minimizing salt accumulation.

**Figure 7:**
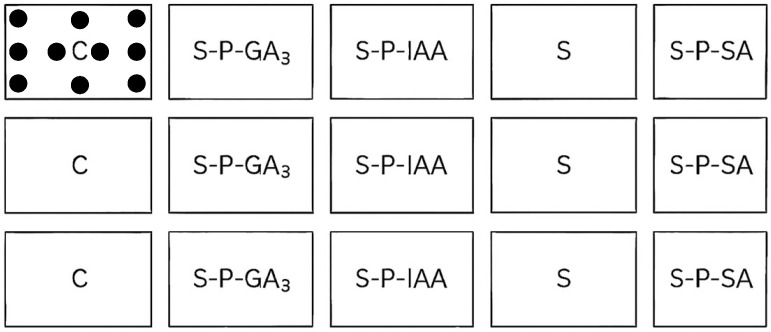
Schematic diagram of the container layout. C: Control, irrigated with tap water; S: salinity stress, irrigated with 5 g L^−1^ NaCl; S-P-IAA: salinity + IAA-primed seeds; S-P-GA3: salinity + GA_3_-primed seeds; and S-P-SA: salinity + SA-primed seeds. The black dots represent the measurement or harvesting sites. The same order of measurements is maintained for all containers.

Containers were arranged according to a completely randomized design (CRD, Figure 7). Treatments were randomly assigned to containers to minimize environmental heterogeneity effects, and measurements, as well as harvesting, were carried out on 10 plants per container, distributed over the entire surface of the container, for a total of 30 replicates per treatment.

### 4.2. In Situ Measurements

At the full vegetative stage (before the reproductive stage, 5-month-old plants), non-destructive measurements (SPAD index and chlorophyll fluorescence) were made. To quantify plant biomass, ten plants were harvested carefully from each plot (30 plants per treatment) and separated into shoots and roots, weighed, and dried at 70 °C for 72 h to determine dry weight. The same pots were maintained with the same treatments until the ears and grains ripened (8-month-old plants). Plant height was measured before harvesting.

#### 4.2.1. SPAD Index

The SPAD index (Soil–Plant Analysis Development) is a non-destructive measurement of leaf greenness, reflecting relative chlorophyll content using a handheld device that measures light absorption at red (650 nm) and infrared (940 nm) wavelengths. The unit SPAD-502 (Konica Minolta, Japan) was used, and measurements were made before the fluorescence measurements on the median fully expanded leaves. Ten plants from each container were used (30 plants per treatment). Values are expressed as SPAD units.

#### 4.2.2. Chlorophyll Fluorescence

Chlorophyll fluorescence measurements were performed using a portable pulse-amplitude modulated fluorometer OS-30p+ (Opti-Sciences, Inc., 8 Winn Avenue, Hudson, NH, USA). Prior to each measurement session, the instrument was allowed to warm up for at least 10 min and was calibrated according to the manufacturer’s instructions, including verification of detector stability and adjustment of the zero-offset using the internal dark reference to ensure accurate baseline fluorescence (F_0_) readings. For the determination of the maximum quantum efficiency of PSII (Fv/Fm), leaves were dark-adapted for 30 min using light-exclusion clips to allow complete oxidation of the PSII reaction centers. Minimum fluorescence (F_0_) was recorded under a weak modulated measuring beam (<0.03 µmol m^−2^ s^−1^), followed by the application of a saturating pulse of approximately 3500 µmol m^−2^ s^−1^ for 0.8 s to obtain maximum fluorescence (Fm). To minimize variability related to leaf age and position, measurements were standardized by selecting the median fully expanded leaf, avoiding the midrib and major veins. The area of interest used for analysis was selected in a circular shape in the center of the leaves. This central part is more homogeneous in thickness and structure. It better reflects actual photosynthetic activity, average stomatal density, chlorophyll content, and tissue moisture status. Measurements were performed with 10 replicates per container for a total of 30 replicates per treatment. Leaves were dark-adapted for 30 min prior to measurements. After measuring the initial Chl, a fluorescence parameter (dark-adapted plants), the plants were exposed to sunlight, and then the same parameters were measured. All measurements were conducted between 09:00 and 11:30 a.m. under stable environmental conditions. The following parameters were measured and presented:

Fv/Fm: maximum quantum yield of PII = (Fm − F0)/Fm,

where

- F0 is the minimum fluorescence excited by a modulated low-intensity red light (0.03 μmol m^−2^ s^−1^);
- Fm is the maximum fluorescence obtained with a pulse of 0.8 s of saturating actinic light (>6000 μmol m^−2^ s^−1^).

YII: The estimated photochemical efficiency index = (Fm′ − Fo)/Fm′,

where F′m is the maximal fluorescence yield of illuminated sample with all PS II centers closed (calculated according to Kramer et al. [98] and Klughammer et al. [99]).

qP: Photochemical quenching = (Fm′ − Fs)/(Fm′ − Fo′)

NPQ: Non-photochemical quenching = (Fm − Fm′)/Fm′

Yloss: Total energy loss = 1 – YII

ETR: The apparent electron transport rate (ETR) according to Bilger et al. [100];

ETR = YII × PFD × 0.5 × 0.84,

where

- PFD is the photon flux density (461 μmol m^−1^ s^−1^) incident on the leaves;
- The value 0.5 is the percentage of the excitation energy distributed to PSII;
- The value 0.84 is the percentage of incident light absorbed by the leaves.

##### Stress Index (SI) Calculation

The stress index (SI) was calculated to quantify the relative reduction in a given parameter under stress conditions compared to the non-stressed (control). It was determined according to the following equation:

SI = 1 − (Xs/Xc)

where

- X = the measured trait (plant height, SI-PltH; dry biomass, SI-DW; SPAD index, SI-Spad; maximum quantum yield of PSII, SI-Fv/Fm; ear filling, SI-EF; and mean weight 100 grains, SI-MW100G);
- Xs = mean value of the measured trait under stress conditions (mean of *n* = 30 plants, 3 repletions of 10 plants for SI-PltH, SI-DW, SI-Spad, and SI-Fv/Fm; or 100 ears, five replicates of twenty ears for SI-EF and SI-MW100G);
- Xc = mean value of the corresponding trait under control (non-stressed) conditions (mean of *n* = 30 plants, 3 repletions of 10 plants for SI-PltH, SI-DW, SI-Spad, and SI-Fv/Fm; or 100 ears, five replicates of twenty ears for SI-EF and SI-MW100G).

The SI expresses the proportional decrease induced by stress relative to the control. Values close to 0 indicate low stress impact (minimal reduction), whereas values approaching 1 indicate a strong negative effect of stress.

##### Artificial Intelligence Use

We used [OREATE AI program, free version] to assist with [graphical abstract generation]. All AI outputs were reviewed and revised by the authors, who take full responsibility for the content

##### Statistical Analysis

Data and statistical analyses were performed using the software XLSTAT (2025.1). All data are presented as mean ± standard error. Analysis of variance (ANOVA) was performed on a mixed-model approach (3 experimental units, containers, of 10 replications) to check whether the effects of the treatment (C, S, S-P-IAA, S-P-GA_3_, S-P-SA) on the respective factors were significant. The significance of differences among treatments was determined by Fisher’s Least Significant Difference test (LSD) at 5%. Means were declared significantly different when the difference between any two treatments was more important than the LSD value generated from the ANOVA. They are marked by different letters in the figures and tables.

## 5. Conclusions

**Figure 8:**
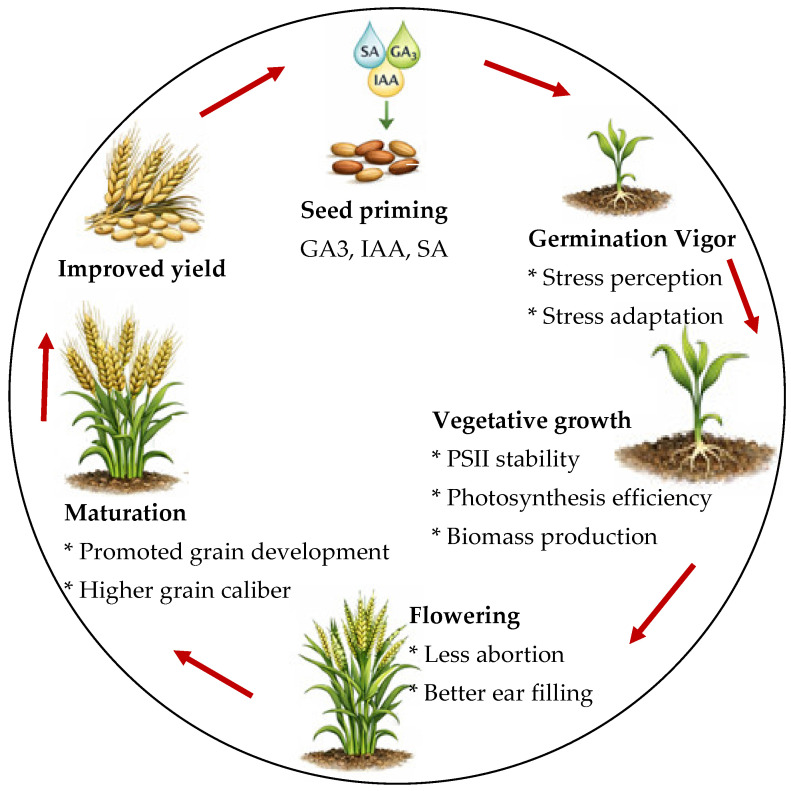
Graphical conclusion summarizing the overall mechanism of hormones used as priming agents on the different stages of the durum wheat life cycle. “Proposed mechanisms are based on observed physiological correlations; direct molecular validation is planned for future work”.

The present study demonstrates that hormonal priming enhances durum wheat performance under moderate salinity stress by promoting a coordinated physiological response that extends from germination to grain maturation. It presents a novel comparative analysis of IAA, GA_3_, and SA seed priming throughout the entire life cycle of durum wheat under moderate salinity, highlighting the distinctive ability of SA to maintain PSII functionality and effectively sustain yield performance. Despite the overall beneficial effects of GA_3_ and IAA, SA appears to be the most efficient priming agent. We suggest that SA stimulates metabolic activity during germination, which leads to improved seed vigor and early establishment; these improvements subsequently support enhanced vegetative growth, biomass accumulation, and sustained photosynthetic efficiency by preserving the integrity and functionality of PSII. We also suggest that this improved physiological status contributes to better reproductive success, reduced floral abortion, improved ear filling, and ultimately superior grain development, as reflected by the increased grain size and MW100G. The continuity of these effects throughout the crop life cycle suggests that SA priming acts as a systemic regulator of stress adaptation rather than a stage-specific stimulant. From a practical standpoint, we recommend SA seed priming at 1 mM for 6 h as a simple, low-cost, and easily adoptable intervention for durum wheat cultivation in soils with moderate salinity stress, pending confirmation through multi-location field trials. This approach could be particularly relevant for semi-arid and salt-affected regions, where improving crop resilience is essential for sustainable production. Moreover, the salinity levels tested do not allow precise identification of the threshold beyond which SA priming may cease to provide agronomic benefit, and long-term or transgenerational effects were not evaluated. Future research should therefore determine the soil salinity thresholds at which SA loses effectiveness and clarify whether its benefits decline progressively or abruptly under severe ionic and osmotic stress. Figure 8 summarizes the overall mechanism of hormonal priming action alongside the life cycle of durum wheat.

## Figures and Tables

**Table 1:** Stress index calculated based on plant height (SI-PltH), dry biomass (SI-DW), SPAD index (SI-Spad), maximum quantum yield of PSII (SI-Fv/Fm), ear filling (SI-EF), and mean weight of 100 grains (SI-MW100G). Fisher’s Least Significant Difference (*n* = 30 plants, three replicates of ten plants each for SI-PltH, SI-DW, SI-Spad, and SI-Fv/Fm; and 100, five replicates of twenty ears for SI-EF and SI-MW100G) was used to test the significance of the means ± standard errors differences. Within rows, the same letter indicates that the means are not significantly different at α = 0.05 (*p* < 0.05).

	SI-PltH	SI-DW	SI-Spad	SI-Fv/Fm	SI-EF	SI-MW100G
S	0.280 ^a^ ± 0.02	0.236 ^a^ ± 0.019	0.184 ^a^ ± 0.010	0.159 ^a^ ± 0.010	0.498 ^a^ ± 0.031	0.344 ^a^ ± 0.025
S-P-IAA	0.236 ^b^ ± 0.017	0.164 ^c^ ± 0.011	0.143 ^b^ ± 0.009	0.085 ^b^ ± 0.007	0.250 ^c^ ± 0.020	0.239 ^bc^ ± 0.019
S-P-GA3	0.198 ^c^ ± 0.011	0.198 ^b^ ± 0.012	0.145 ^b^ ± 0.008	0.036 ^c^ ± 0.002	0.299 ^b^ ± 0.022	0.258 ^b^ ± 0.021
S-P-SA	0.100 ^d^ ± 0.007	0.100 ^d^ ± 0.008	0.026 ^c^ ± 0.002	0.027 ^d^ ± 0.002	0.168 ^d^ ± 0.011	0.091 ^c^ ± 0.007

SI = 1 − (Xs/Xc). X_s_ = mean value of the measured trait under stress conditions (S, stress). X_c_ = mean value of the measured trait under non-stress conditions (C, control). Values closer to 0 indicate minimal stress impact.

**Table 2:** Pearson correlation matrix. DW: Dry weight; EF: ear filling; ETR: electron transport rate; Fv/Fm: maximum quantum yield of PSII; MW100G: mean weight of 100 grains; Plt-H: plant height; Spad: SPAD index; YII: effective quantum yield of PSII.

Variables	Fv/Fm	YII	ETR	DW	Plt-H	SPAD Index	EF	MW100G
Fv/Fm	1	0.674	0.672	0.777	0.834	0.745	0.891	0.805
YII	0.674	1	1.000	0.893	0.892	0.972	0.877	0.948
ETR	0.672	1.000	1	0.893	0.891	0.972	0.876	0.947
DW	0.777	0.893	0.893	1	0.966	0.952	0.961	0.984
Plt-H	0.834	0.892	0.891	0.966	1	0.970	0.933	0.982
Spad	0.745	0.972	0.972	0.952	0.970	1	0.913	0.988
EF	0.891	0.877	0.876	0.961	0.933	0.913	1	0.962
MW100G	0.805	0.948	0.947	0.984	0.982	0.988	0.962	1

Note: ETR is calculated from YII (see Methods), explaining the perfect correlation between these variables.

## Data Availability

The original contributions presented in this study are included in the article. Further inquiries can be directed to the corresponding author.

## Abbreviations

The following abbreviations are used in this manuscript:

ABA	Abscisic acid
DW	Dry weight
EF	Ear filling
ETR	Electron transport rate
Fv/Fm	Maximum quantum yield of PSII
GA3	Gibberellic acid
IAA	Indole-3-acetic acid
MW100G	Mean weight of 100 grains
Plt H	Plant heigh
qP	Photochemical quenching
NPQ	Non-photochemical quenching
SA	Salicylic acid
SI	Stress index
Yloss	Total energy loss
YII	Estimated photochemical efficiency index
